# Changing local recombination patterns in Arabidopsis by CRISPR/Cas mediated chromosome engineering

**DOI:** 10.1038/s41467-020-18277-z

**Published:** 2020-09-04

**Authors:** Carla Schmidt, Paul Fransz, Michelle Rönspies, Steven Dreissig, Jörg Fuchs, Stefan Heckmann, Andreas Houben, Holger Puchta

**Affiliations:** 1 Botanical Institute, Karlsruhe Institute of Technology, Fritz-Haber-Weg 4, 76133 Karlsruhe, Germany; 2 Department of Plant Development and (Epi)Genetics, Swammerdam Institute of Life Sciences, University of Amsterdam, Postbus 1210, 1000 BE Amsterdam, Netherlands; 3 Institute of Agricultural and Nutritional Sciences, Martin Luther University Halle Wittenberg, Karl-Freiherr-von-Fritsch-Str. 4, 06120 Halle, Germany; 4 Leibniz Institute of Plant Genetics and Crop Plant Research, Corrensstraße 3, 06466 Gatersleben, Germany

**Keywords:** Genetic engineering, Molecular engineering in plants, Plant molecular biology

## Abstract

Chromosomal inversions are recurrent rearrangements that occur between different plant isolates or cultivars. Such inversions may underlie reproductive isolation in evolution and represent a major obstacle for classical breeding as no crossovers can be observed between inverted sequences on homologous chromosomes. The heterochromatic knob (hk4S) on chromosome 4 is the most well-known inversion of Arabidopsis. If a knob carrying accession such as Col-0 is crossed with a knob-less accession such as L*er*-1, crossovers cannot be recovered within the inverted region. Our work shows that by egg-cell specific expression of the Cas9 nuclease from *Staphylococcus aureus*, a targeted reversal of the 1.1 Mb long hk4S-inversion can be achieved. By crossing Col-0 harbouring the rearranged chromosome 4 with L*er*-1, meiotic crossovers can be restored into a region with previously no detectable genetic exchange. The strategy of somatic chromosome engineering for breaking genetic linkage has huge potential for application in plant breeding.

## Abstract (web summary)

The heterochromatic knob (hk4S) on Arabidopsis chromosome 4 prevents the recombination between accessions with and without hk4S. Here, via egg-cell specific expression of the Cas9 nuclease, the authors demonstrate targeted reversal of the 1.1 Mb long hk4S-inversion in Col-0 and restore the crossovers with L*er*-1.

## Introduction

The variability of genetic information in every organism serves as a basis for the broad biodiversity within our ecosystem. Besides simple mutations leading to alterations of one or more bases, changes of chromosome structure can be repeatedly observed during the evolution of different species^1–4^. In particular, a change in the orientation of chromosome segments, such as inversions, is associated with speciation, adaption, and genome evolution^5–8^. It was shown for plants that quantitative trait loci (QTLs) can be located in inverted regions^9–11^, which can cause serious problems if certain genes should be transferred between different cultivars by crossing^12,13^. By the use of adaptable site-specific nucleases, it is possible to modify the structure of plant chromosomes in a targeted manner^14–16^. Thus, very recently we could demonstrate that arms can be exchanged between heterologous chromosomes in Arabidopsis in reciprocal manner^17^. Moreover, we were able to show that by the use of the CRISPR/Cas system, in principle, it is possible to invert chromosomal sequences within the kb range in Arabidopsis by the specific induction of two DSBs using egg cell specific expression^18^ of the *Staphylococcus aureus* (SaCas9)^19^ nuclease^20^. However, to restore recombination in naturally rearranged areas of the chromosome, heritable inversions within the Mb range are required.

Upon comparison of the genomic sequences of the two Arabidopsis ecotypes Columbia (Col-0) and Landsberg *errecta* (L*er*-1), a number of inversions can be detected^21^. The best known of these is the hk4S inversion, which has a size of 1.17 Mb that led to a shift of a pericentromeric, heterochromatic knob region into the middle of the short arm of chromosome 4. Whereas chromosome 4 in L*er*-1 represents the evolutionary older conformation, the corresponding chromosome 4 of Col-0 carries the hk4S inversion. The heterochromatic knob of Col-0 has been characterized in detail by cytological and sequence-based analyses^22,23^. The exact localization of the two inversion junctions was recently elucidated and a model for the formation of this inversion due to the activity of a vandal-transposable element (TE) type, was suggested^24^. The inversion occurred ~5000 years ago and the resulting progeny was distributed widely. Thus, like Col-0, 170 accessions of Arabidopsis in Europe and North America carry the hk4S inversion, whereas other cultivars besides L*er*-1 do not. Col-0 and L*er*-1 are the two cultivars most widely used in genetic studies for mapping due to the presence of multiple SNPs between them. In the context of recombination analyses of F1-hybrids of the two cultivars, it was demonstrated that no crossovers (COs) were observed within the entire inverted chromosome region^25,26^. Here, we describe our process of reverting the hk4S inversion in Col-0 to enable COs to be recovered between the cultivars in the respective region using the highly efficient Cas9 nuclease from *Staphylococcus aureus*.

## Results

### Identification of suitable protospacer sequences

**Fig. 1:**
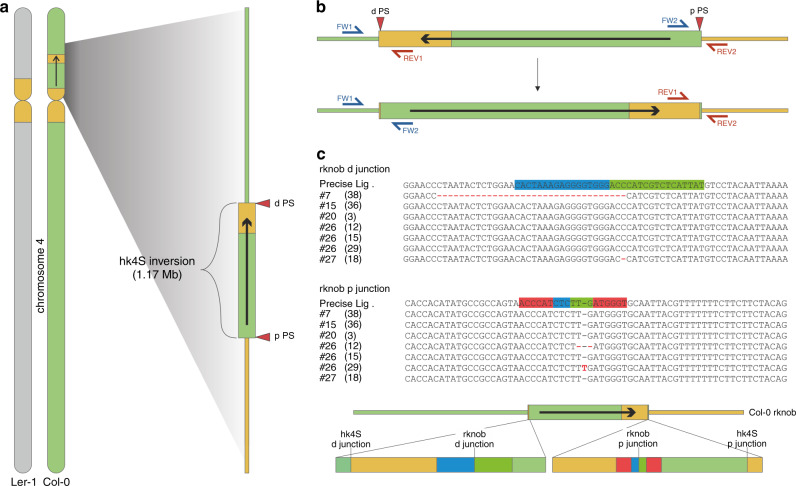
Design, detection and composition of junctions of the induced hk4s reversion. **a** Overview of chromosome 4 in Col-0 (light green) and L*er*-1 (gray). The inversion in Col-0 is located on the short chromosome arm and thereby part of the pericentromeric region (orange) is shifted into the middle of this chromosome arm. The location of both protospacers, distal (d PS) and proximal (p PS) relative to the centromere, are marked as red triangles. **b** Schematic representation of the detection of the inversion after DSB induction. Thus, the formerly inverted region is reverted. The induced inversion can be detected using site-specific primers for both junctions. Primers FW1 and FW2 (blue) are specific for the p junction, and primers REV1 and REV2 (red) are specific for the d junction. **c** Sequence analysis of both newly formed junctions reveals seven independent heritable induced inversion events referred to as reverted knob (rknob) inversions. The sequence of the d protospacer is depicted in blue, the sequence of the p protospacer is depicted in green, and PAM sequences are marked in red. Source data are provided as a Source Data file.

As a first step, we used the available sequence information^24^ to identify suitable protospacer sequences located closely to both original inversion junctions. Furthermore, we ensured that the chosen protospacers (named proximal (p) and distal (d) protospacer (PS), according to their orientation to the centromere) were located in intergenic regions in order to avoid disturbance of surrounding genes (Fig. 1a). Both protospacers used were placed in the kb range close to the original hk4S d junctions (for spacer sequences see Supplementary Table 1). These protospacers were cloned into the pDe-Sa-Cas9 vector with Sa-Cas9 under the control of the egg cell-specific EC1.1/EC1.2-promotor^18^. The vector construct is depicted in Supplementary Fig. 1 and it was finally transformed into *A. thaliana* Col-0 wild type plants via floral-dip.

### Identification of plants harbouring the inversion

Transgenic T_1_ seeds were selected and grown until seed set and we obtained seeds from 38 primary transformants. From these, 40 individual T_2_ plants were grown for each individual T_1_ line for 2 weeks on GM medium without selection. Leaf material from each of the 40 T_2_ siblings was pooled and DNA of these bulk samples, each representing the progeny of an individual T_1_ line, was extracted. The detection of a chromosome inversion within the respective pools was carried out with the help of junction-specific primer combinations (Fig. 1b). The results of PCR-based genotyping are shown in Table 1. In ten of the 38 analyzed pools, specific bands for both newly formed junctions of the inversion could be detected. Out of this, five pools were analyzed further by taking leaf material from the 40 individual plants and isolating DNA. In all five pools, plants with a heritable inversion event could be detected. For three T_1_ lines (#7, 15, 27) one plant each carrying the inversion, and for another T_1_ line (#26) three independent plants with an inversion could be isolated. As in the case of line #20, most plants (34 of 40) were positively tested for the inversion and we speculated that the inversion occurred in the egg cell during transformation. This would result in a T_1_ plant heterozygous for the induced inversion with progeny that is segregating in the T_2_ generation. Indeed, screening simultaneously more than 200 T_2_ seedlings for the original, as well as the new junctions, revealed proper Mendelian segregation (Supplementary Table 3) confirming our hypothesis. This enabled us to isolate a large number of plants in the T_2_ that contained the induced inversion in a homozygous state. In addition, T_2_ plants representing independent inversion events (out of T_1_-lines #7, 15, 26 (12), 26 (19), 27, only #26 (15) could not be examined because the plant died before seed set in the greenhouse) were further propagated and tested for segregation of the inversion (see Supplementary Table 4). Each of the five tested lines showed proper Mendelian segregation of the inversion in T_3_, which enabled us to identify several plants per line harbouring the induced inversion in a homozygous state. The Phenotype and the fertility of plants carrying the hk4S reversion did not differ from wild type plants. To sum up, using our setup we were able to achieve the heritable reversion of the hk4S knob and obtained six different heritable inversion events, resulting in a successful reversion of the hk4S knob.

**Table 1:** List of induced inversions detected in the T_2_ generation as determined by PCR-based genotyping.

Tested lines	Positive lines	Detailed analyzed T_2_ lines	Tested T_2_ plants	Positive rknob plants
38	10	# 7	40	1
		# 15	40	1
		# 20	40	34
		# 26	40	3
		# 27	40	1

Five out of ten positive tested T_2_ lines were analyzed in detail and each of these lines showed at least one positive plant carrying the reverted knob (rknob) inversion.

### Molecular characterization of the inversion

We elucidated the molecular nature of the newly formed junctions by PCR and sequencing in T_2_. The analysis revealed that the inversion occurring in the T_1_ plant of line #20 is due to a re-ligation of the broken ends without any sequence loss. The same holds true for the T_2_ event isolated from line #15 and #26 (15). In the case of line #7, the newly formed d junction carries a deletion of 34 bp whereas the p junction was due to simple ligation. In the case of line #27, the p junction arose due to precise ligation whereas the d junction contained a 1 bp deletion. Most interestingly, the junctions of all three events isolated from line #26 differed, which can be taken as strong evidence that all three events arose independently. Here in all cases, the d junction arose due to simple ligation as the p junction of #26 (15), whereas the sequences of the p junctions of #26 (12) and #26 (29) differed, containing a 2 bp deletion and a 1 bp insertion (Fig. 1d). Thus, in the majority of cases, the inversion does not result in any sequence loss (10 out of 14 junctions). To make sure that no significant parts of the inverted area were deleted during the formation of the newly induced inversion, the DNA of the line #20, homozygous for the inversion, was analyzed further. We scanned for the presence of the inverted region by PCR, amplifying equally distributed 1–2 kb long fragments for each 100 kb (Supplementary Fig. 2, Supplementary Table 5) and found no indication for any sequence loss. To characterize further the induced inversion, the homozygous line #20, was analyzed via FISH using already established BAC clones of Fransz et al.^24^.

**Fig. 2:**
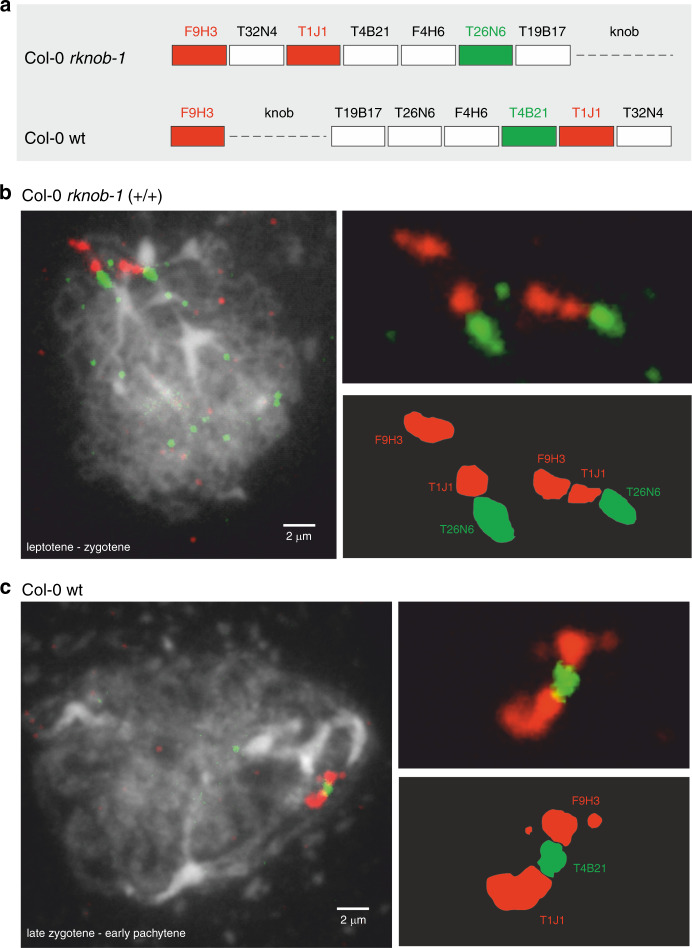
Fluorescence in situ hybridization to Col-0 WT and the homozygous *rknob-1*. **a** Schematic overview of the expected fluorescence patterns of inversion BACs relative to the reference F9H3, which maps distal from the knob hk4S. Two independent FISH experiment were performed for both Col-0 and rknob-1. In both cases the same fluorescence pattern were obtained as shown in the figure as well as in the Source Data file. **b** Early zygotene meiocyte of *rknob-1* showing red-red-green signals in both homologues of chromosome 4. The length of the scale bar is 2 µm. **c** Prophase I meiocyte of Col-0 WT with red-green-red signals. The length of the scale bar is 2 µm. Source data are provided as a Source Data file.

FISH was carried on to Prophase I meiocytes, in which the chromosomes are visible as individual, condensed chromatin fibers. We used the BACs T1J1, T26N6, and T4B21 that are mapped in the inversion region and BAC F9H3, mapped distal from the knob hk4S, as reference. The fluorescent detection of the labeled BAC probes was chosen to distinguish the inverted from the reverted orientation (Fig. 2a). The Col-0 preparations all showed the BAC order F9H3 – T4B21 – T1J1, indicating the inverted orientation. The line *rknob-1*, which (Fig. 2b) is homozygous for the newly induced inversion, clearly shows a reversed order of the BACs (F9H3 – T1J1 – T26N6), indicating that the region is reverted (Fig. 2c).

### Restoration of meiotic recombination within the inversion

**Fig. 3:**
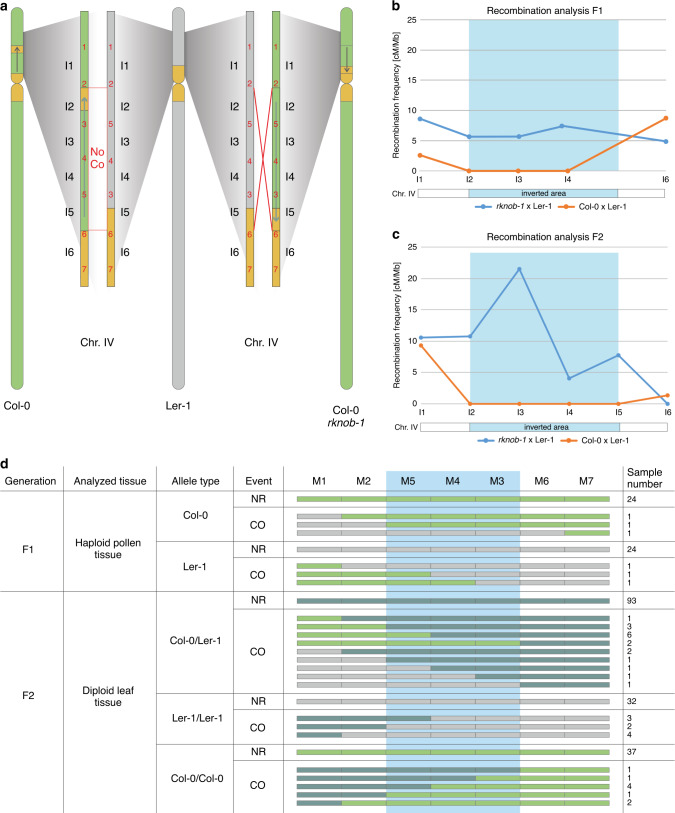
SNP-marker based recombination analysis in F1- and F2-hybrids. **a** Overview of SNP-marker (red) distribution on chromosome 4 in Col-0 (green), *rknob-1* and L*er*-1 (gray) spanning the inversion and both border regions. Two SNP-markers form one interval resulting in six different intervals (I1-I6). **b** Results of recombination analysis based on SNP genotyping of pollen nuclei in F1-hybrids. Fifty-two samples were used for *rknob-1* × L*er*-1 and 90 samples for the control. Recombination frequencies are given as cM/Mb, with the amount of CO being set in relation to the distance between the two corresponding markers. The inverted area is highlighted in light blue in the diagram. **c** Results of recombination analysis based on SNP genotyping of leaf material of F2-hybrids. 198 samples were analyzed for *rknob-1* × L*er*-1 and 200 samples for the control. Recombination frequencies are given as cM/Mb, with the amount of CO being set in relation to the distance between the two corresponding markers. The inverted area is highlighted in light blue in the diagram. **d** Detailed overview of composition and amount of detected CO and no recombination (NR) events in haploid pollen tissue (F1) and diploid leaf tissue (F2) in the *rknob-1* × L*er*-1 line. Detection of Col-0 allele is marked in light green, detection of L*er*-1 allele is marked in gray, and when both alleles were detected the marker is shown in dark green. The inverted area is highlighted in light blue. Source data are provided as a Source Data file.

In a subsequent step, we tested whether we are able to reactivate recombination in the formerly CO dead region in a cross with the L*er*-1 by the CRISPR/Cas induced inversion of the hk4S knob. We crossed the homozygous line #20, now referred to as *rknob-1*, with L*er*-1. As a control, we used Col-0 wild type plants crossed with L*er*-1. Recombination frequencies were determined via SNP based genotyping via the use of seven markers to discriminate between Col-0 and L*er*-1, (Fig. 3a). Marker 2 and Marker 6 are located outside of the inversion but in direct vicinity to the inversion junctions with a distance of only a few hundred base pairs, representing both borders of the inversion.

Initially, we analyzed haploid pollen for COs within the inverted region. The F1-hybrids resulting from crossings were grown until flowering whereby the inflorescences were collected^27,28^. Pollen nuclei were isolated, separated via FACS and finally whole-genome amplification was performed^29,30^. As a quality control, we tested for the presence of three different areas harboring SNP-markers to identify successfully amplified pollen DNA. Then, we determined CO frequencies for the five intervals (see Fig. 3a) and the results are pictured in Fig. 3b as recombination frequencies relative to the distance between both markers. We were able to detect three independent CO events equally distributed over the inverted area (Fig. 3d) in 54 samples of the inversion line (*rknob-1* × L*er*-1), whereas no CO event could be detected within the inverted area in 90 samples of the control.

To confirm these results for the offspring and to increase sample size, seeds from the hybrids were harvested and the respective F2-plants were again tested for CO formation via SNP based genotyping for six intervals. All in all, 198 individual F2 plants of the *rknob-1* × L*er*-1 line and 200 individual F2 plants of the control were tested using the same primers and probes as in F1-analysis. CO frequencies were determined and results are shown in Fig. 3c. Again, we did not detect any CO formation within the inversion (I2-I5) in the control, while restoration of recombination within the inversion in the *rknob-1* × L*er*-1 line was observed. Outside the inversion, both lines showed a comparable recombination rate with a clear difference with the euchromatic areas in the middle of the chromosome arm (I1) showing higher recombination frequencies than in the pericentromeric region (I6). Overall, we detected in the *rknob-1* × L*er*-1 line nine CO events in I1, 27 CO events within the inversion and no CO event near the centromere (I6) (Fig. 3d). Thus, by reverting the knob inversion we were able to restore CO formation in a formerly inaccessible part of chromosome 4 and changed recombination patterns.

## Discussion

Here we were able to show that by the use of CRISPR/Cas, chromosomal inversions in the Mb range can be achieved precisely and efficiently in plants. We were able to obtain seven independent Mb-sized inversion events by screening the progeny of 38 primary transformants, resulting in an overall frequency of about 0.5%. Due to a timesaving pooling approach^20^, instead of over 1500 samples, <250 samples had to be analyzed to achieve this goal. An increase of frequencies for further application in crops could be achieved by a knock-out of classical NHEJ factors like KU70. We could show that loss of KU70 results in a significant increase of inversion as well as chromosomal translocation frequencies, although this is associated with a loss of accuracy^17,20^. We were surprised that we were able to obtain the 1.17 MB spanning knob reversion at only marginally lower frequencies than inversions spanning 3–18 kb. This indicates that there is no linear correlation between inversion size and inversion frequency^20^. It might well be that different broken ends are clustered during repair in the plant nucleus, explaining this lack of correlation with size. But to conclusively address this question, further experiments with different inversions sizes need to be obtained in future.

We regard our finding that we were able to awake a CO dead region by reverting a natural inversion that arose during plant evolution as of central importance for plant breeders. Many crop plants carry inversions, which have occurred naturally over time as a result of erroneous repair processes^3,31,32^. Recent improvements in sequence analysis of crop plants such as barley reveal that multi-mega base inversions occur with high frequency between different genotypes^12^, leading to CO suppression and it was also observed that CO rates are slightly elevated nearby rearranged areas^33^. Loss of COs in inverted areas can inhibit the transfer of certain QTLs or resistance markers between different crop cultivars. For example, recombination in the region of the nematode resistance gene *Mi-1*, which is associated with an inverted chromosome segment in *Lycopersicon peruvianum*, is strongly suppressed when crossed with the susceptible cultivar *Lycopersicon esculentum*^34^. By reverting a naturally arosen inversion in *Arabidopsis thaliana* we were able to show that it is now possible to solve this kind of problem in plants with reasonable efficiency. Using CRISPR/Cas, we should not only be able to revert natural inversions and thus target COs in recombination dead regions, but we should also be able to achieve linkages between attractive traits by creating inversions, leading to substantial change in recombination patterns in plants.

Beside inversions, translocations are the main driving force of genome evolution on the chromosomal level^1,2^. We could just demonstrate that chromosomal translocation can be achieved using CRISPR/Cas technology in plants, too. We were able to obtain heritable reciprocal chromosome arm exchanges between chromosome 1 and 2 and 1 and 5 in Arabidopsis^17^. Thus, it is possible to induce Mbpp large changes not only within but also between chromosomes. Using both approaches different kinds of genetic linkages can be stabilized or broken. With these tools in hand plant genome editing has raised to a new level, chromosome structural engineering^35^. It will now be important to adopt the technology to the most important crop plants to use its full potential for revolutionizing breeding.

## Methods

### Cloning of T-DNA constructs

Cloning of DNA constructs was done using the plasmids pDe-Sa-Cas9 and pEn-Sa-Chimera^19,36^. However, using the restriction enzymes PmeI and SbfI, the kanamycin resistance cassette was replaced by a bar resistance cassette. Spacer sequences for the p and the d junction were cloned into individual pEn-Sa-Chimera vectors. Together they were integrated into pDe-Sa-Cas9. The corresponding spacer sequences that are specific for both borders of the hk4S knob are listed in Supplementary Table 1. Using Bsu36I and MluI, the first chimera was added. The second chimera was transferred via a Gateway^®^ LR-reaction. The EC1.1-promoter (composed of the EC1.1 promoter combined with the EC1.2 enhancer)^18^ and the rbcS-E9 terminator were used for egg cell specific expression. Both fragments were amplified from pHEE2E-TRI, and via Gibson assembly^®^ (New England Biolabs, NEB, https://www.neb.com/) and inserted into the respective pDe-Sa-Cas9 vector. Primers used for cloning are listed in Supplementary Table 1.

### Plant transformation and growth conditions

*Arabidopsis thaliana* cultivars used in this study were in the Columbia (Col-0) and Landsberg errecta (L*er*-1) background. The plants were cultivated either in a growth chamber or in the greenhouse at 22 °C with 16 h light and 8 h darkness. In the growth chamber, agar plates were used containing germination medium (GM: 4.9 g/l Murashige & Skoog-medium, 10 g/l saccharose, pH 5.7, 7.6 g/l plant-agar) and in the greenhouse cultivation was conducted on substrate containing 1:1 mixture of Floraton 3 (Floragard, Oldenburg, Germany) and vermiculite (2–3 mm, Deutsche Vermiculite Dämmstoff, Sprockhövel, Germany). Arabidopsis lines were transformed with the *A. tumefaciens* strain GV3101 via the floral dip method^37^.

### DNA extraction

For this study, DNA was extracted via a shortcut DNA extraction method. Therefore, DNA material was grinded using a pestle suitable for 1.5 mL reaction tubes, to which 500 µL shorty extraction buffer (200 mM Tris-HCl (pH 9), 400 mM LiCl, 25 mM EDTA, 1% SDS, pH 9,0) was added. The mixture was then centrifuged for 5 min at 17,000 *g* and 400 µL of the supernatant was mixed with 400 µL 2-propanol. After the sample was thoroughly inverted, the DNA was pelleted for 10 min at 19,500 *g* in a further centrifugation step. After the supernatant was removed, the pellet was dried and then dissolved in TE buffer (10 mM Tris-HCl (pH 9,0), 1,0 mM EDTA, pH 8,0).

### Detection and analysis of heritable reverted inversions

The detection of heritable reverted inversions was carried out according to the already established protocol of Schmidt et al. 2019^20^. T_2_ seeds of T_1_ plants with a stable integrated T-DNA were cultivated on GM medium in a growth chamber for 2 weeks. Subsequently, a collective DNA extraction of each individual line was executed and these bulk samples were screened for successful reversal of the inversion via PCR. Sequencing of junctions was performed by GATC Eurofins and ApE (v2.0.55) was used for alignment and analysis of Sanger sequencing data. Up to five positively tested lines were then used to identify the individual plants harboring the reverted inversion. Therefore, leaf material of 40 individual plants was collected and after DNA extraction, the individual samples were screened via PCR for both newly formed junctions. The positive plants were grown until the seeds ripened and these T_3_ seeds were then tested for Mendelian segregation and the identified homozygous plants were further propagated. Finally, the respective T_4_ plants were re-tested for their homozygous status and then used for further analyses.

### Sorting of haploid nuclei and whole-genome amplification

Single pollen nuclei DNA was extracted from the F1 seeds derived by crossbreeding. The plants were grown until flowering and 15–20 inflorescences were collected in 1.5 mL Eppendorf tubes. 300 µL ddH_2_O were added and vortexed for ~30 s. For the release of pollen grains the suspension was shaken at 150 rpm for 10 min at room temperature. Afterwards, the suspension was centrifuged at 17,000 *g* for 5 min. After manual removing the empty anthers the suspension was centrifuged again at 17,000 *g* for 5 min. The supernatant was discarded and the pellet was then resuspended in 100 µL Galbraith buffer (45 mM MgCl2, 30 mM sodium citrate, 20 mM MOPS, 0.1% TritonX100, pH to 7.0). The suspension was transferred into a 2 mL Eppendorf tube containing two metallic beads of 6 mm diameter (Intec GmbH). After a further centrifugation step at 17,000 *g* for 5 min, the suspension was homogenized at 30 Hz for 40 s using a MM 400 ball mill (Retsch). After adding 500 µL Galbraith buffer the suspension was filtered through a 30 µm filter (Sysmex-Partec). For staining 4′,6-diamidino-2-phenylindole (DAPI; 1.5 μg/mL) was used. Using a BD Influx Cell Sorter (BD Biosciences) single 1 C nuclei were sorted into individual wells of a 384-microwell plate. Each well contained 2 μL lysis solution (0.5 μL lysis buffer composed of 400 mM KOH, 100 mM DTT, 10 mM EDTA; 0.5 μL ddH2O; and 1 μL sample buffer (Genomiphi V2, GE Healthcare)) for whole-genome-amplification. The sample buffer containing random primers for whole-genome amplification was added to lysis solution. Using the Genomiphi V2 kit (GE Healthcare) whole-genome-amplification was carried out. However, the manufacturer’s protocol was modified in the following way: By incubation at 65 °C for 3 min in 2 µL lysis solution nuclei lysis and DNA denaturation was conducted. By adding 0.5 µL neutralization buffer (666 mM Tris-HCl, 250 mM HCl^15^) the lysis solution was neutralized. As next step, a master mix composed of 3.5 µL sample buffer, 4.5 µL reaction buffer, and 0.5 µL enzyme mix (Genomiphi V2, GE Healthcare) per reaction was added. After incubation at 30 °C for 8 h the enzyme was inactivated by an incubation step at 65 °C for 10 min^29^. Each sample was diluted with 500 µL ddH_2_O and was used for subsequent SNP genotyping.

### Determination of co-frequency

The CO-frequency was determined by the detection of a marker change via SNP genotyping using cultivar specific probes. Therefore, seven different TaqMan assays with specific primer and probe combinations were designed, spanning the inverted and adjacent regions that are listed in Supplementary Table 1 and 2. The analysis was performed using a Lightcycler^®^ 480 II (Roche) and the PerfeCTa^®^ qPCR ToughMix^®^ (Quantabio) according to the manufacturer’s protocol, but with a total reaction volume of 10 µL. The data were finally evaluated with LightCyler^®^ 480 SW 1.5. Graphs were made with Excel 2016 and CorelDraw 2019 (Version 21.0.0.593).

### Cytogenetic analysis

Fluorescence in situ hybridization was carried out on prophase 1 meiocytes prepared from immature flower buds that were fixed in ethanol/acetic acid (3:1), following the protocol by Ross et al.^38^. The following BAC clones were used: F9H3, T19B17, T26N6, F4H6, T4B21, T1J1 (IGF and TAMU library)^39,40^. The BAC DNA clones were labeled with either digoxigenin-11-dUTP or biotin-16-dUTP (Jena Bioscience GmbH) by nick translation following the manufacturer’s protocol (Sigma-Aldrich). In situ hybridization was carried out according to Lysak et al., 2006 with separate denaturation of probes and chromosomes^41^. Microscopy slides were examined with a Zeiss AxioScope A1 fluorescence microscope using small band pass filters for DAPI, FITC, and Cy3. Images were captured with a Nikon color DS-Ri2 camera using Nikon NIS-elements 4.60 software. Microscope images were further processed with Adobe Photoshop software.

### Reporting summary

Further information on research design is available in the Nature Research Reporting Summary linked to this article.

## Supplementary information

Supplementry Information

Reporting Summary

## Data Availability

Data supporting the findings of this work are available within the paper and its Supplementary Information files. A reporting summary for this Article is available as a Supplementary Information file. The datasets generated and analyzed during the current study are available from the corresponding author upon request. Source data are provided with this paper.

## Source data

Source Data

## References

[1] Schubert I, Vu GTH (2016). Genome stability and evolution: attempting a holistic view. Trends plant Sci..

[2] Alkan C, Coe BP, Eichler EE (2011). Genome structural variation discovery and genotyping. Nat. Rev. Genet..

[3] Fang Z (2012). Megabase-scale inversion polymorphism in the wild ancestor of maize. Genetics.

[4] Wellenreuther M, Bernatchez L (2018). Eco-evolutionary genomics of chromosomal inversions. Trends Ecol. Evol..

[5] Faria R, Johannesson K, Butlin RK, Westram AM (2019). Evolving inversions. Trends Ecol. Evol..

[6] Termolino, P. et al. Recombination suppression in heterozygotes for a pericentric inversion induces the interchromosomal effect on crossovers in Arabidopsis. *Plant J.: Cell Mol. Biol.*10.1111/tpj.14505 (2019). 10.1111/tpj.14505 PMC6973161 31436858

[7] Lysak MA (2006). Mechanisms of chromosome number reduction in Arabidopsis thaliana and related Brassicaceae species. Proc. Natl Acad. Sci. USA.

[8] Rieseberg LH (2001). Chromosomal rearrangements and speciation. Trends Ecol. Evol..

[9] Lee C-R (2017). Young inversion with multiple linked QTLs under selection in a hybrid zone. Nat. Ecol. Evol..

[10] Coughlan JM, Willis JH (2019). Dissecting the role of a large chromosomal inversion in life history divergence throughout the Mimulus guttatus species complex. Mol. Ecol..

[11] Lowry, D. B., Popovic, D., Brennan, D. J. & Holeski, L. M. Mechanisms of a locally adaptive shift in allocation among growth, reproduction, and herbivore resistance in Mimulus guttatus. *Evol.; Int. J. Org. Evol.*10.1111/evo.13699 (2019). 10.1111/evo.13699 30793293

[12] Himmelbach A (2018). Discovery of multi-megabase polymorphic inversions by chromosome conformation capture sequencing in large-genome plant species. Plant J.: Cell Mol. Biol..

[13] Keilwagen J (2019). Detecting large chromosomal modifications using short read data from genotyping-by-sequencing. Front. Plant Sci..

[14] Pacher M, Schmidt-Puchta W, Puchta H (2007). Two unlinked double-strand breaks can induce reciprocal exchanges in plant genomes via homologous recombination and nonhomologous end joining. Genetics.

[15] Schmidt, C., Schindele, P. & Puchta, H. From gene editing to genome engineering: restructuring plant chromosomes via CRISPR/Cas. *aBIOTECH*10.1007/s42994-019-00002-0 (2019). 10.1007/s42994-019-00002-0 PMC9584095 36305002

[16] Siebert R, Puchta H (2002). Efficient repair of genomic double-strand breaks by homologous recombination between directly repeated sequences in the plant genome. Plant Cell.

[17] Beying N, Schmidt C, Pacher M, Houben A, Puchta H (2020). CRISPR–Cas9-mediated induction of heritable chromosomal translocations in Arabidopsis. Nat. Plants.

[18] Wang Z-P (2015). Egg cell-specific promoter-controlled CRISPR/Cas9 efficiently generates homozygous mutants for multiple target genes in Arabidopsis in a single generation. Genome Biol..

[19] Steinert J, Schiml S, Fauser F, Puchta H (2015). Highly efficient heritable plant genome engineering using Cas9 orthologues from Streptococcus thermophilus and Staphylococcus aureus. Plant J.: Cell Mol. Biol..

[20] Schmidt C, Pacher M, Puchta H (2019). Efficient induction of heritable inversions in plant genomes using the CRISPR/Cas system. Plant J.: Cell Mol. Biol..

[21] Zapata L (2016). Chromosome-level assembly of Arabidopsis thaliana Ler reveals the extent of translocation and inversion polymorphisms. Proc. Natl Acad. Sci. USA.

[22] The complete sequence of a heterochromatic island from a higher eukaryote. (2000). The Cold Spring Harbor Laboratory, Washington University Genome Sequencing Center, and PE Biosystems Arabidopsis Sequencing Consortium. Cell.

[23] Fransz PF (2000). Integrated cytogenetic map of chromosome arm 4S of A. thaliana: structural organization of heterochromatic knob and centromere region. Cell.

[24] Fransz P (2016). Molecular, genetic and evolutionary analysis of a paracentric inversion in Arabidopsis thaliana. Plant J.: Cell Mol. Biol..

[25] Drouaud J (2006). Variation in crossing-over rates across chromosome 4 of Arabidopsis thaliana reveals the presence of meiotic recombination “hot spots”. Genome Res..

[26] Giraut L (2011). Genome-wide crossover distribution in Arabidopsis thaliana meiosis reveals sex-specific patterns along chromosomes. PLoS Genet..

[27] Chen P-H, Pan Y-B, Chen R-K (2008). High-throughput procedure for single pollen grain collection and polymerase chain reaction in plants. J. Integr. Plant Biol..

[28] Drouaud J, Mézard C (2011). Characterization of meiotic crossovers in pollen from Arabidopsis thaliana. Methods Mol. Biol. (Clifton, N. J.).

[29] Dreissig S (2015). Measuring meiotic crossovers via multi-locus genotyping of single pollen grains in barley. PloS ONE.

[30] Dreissig S, Fuchs J, Himmelbach A, Mascher M, Houben A (2017). Sequencing of single pollen nuclei reveals meiotic recombination events at megabase resolution and circumvents segregation distortion caused by postmeiotic processes. Front. Plant Sci..

[31] Badaeva ED (2007). Chromosomal rearrangements in wheat: their types and distribution. Genome.

[32] Rodríguez-Leal D, Lemmon ZH, Man J, Bartlett ME, Lippman ZB (2017). Engineering quantitative trait variation for crop improvement by genome editing. Cell.

[33] Rowan BA (2019). An ultra high-density arabidopsis thaliana crossover map that refines the influences of structural variation and epigenetic features. Genetics.

[34] Seah S, Yaghoobi J, Rossi M, Gleason CA, Williamson VM (2004). The nematode-resistance gene, Mi-1, is associated with an inverted chromosomal segment in susceptible compared to resistant tomato.. Theor. Appl. Genet. Theoretische und Angew. Genetik.

[35] Lee, K. & Wang, K. Level up to chromosome restructuring. *Nat. Plants*. 10.1038/s41477-020-0669-4 (2020). 10.1038/s41477-020-0669-4 32451450

[36] Steinert J, Schmidt C, Puchta H (2017). Use of the Cas9 Orthologs from Streptococcus thermophilus and Staphylococcus aureus for Non-Homologous End-Joining Mediated Site-Specific Mutagenesis in Arabidopsis thaliana. Methods Mol. Biol. (Clifton, N. J.).

[37] Clough SJ, Bent AF (1998). Floral dip: a simplified method for Agrobacterium-mediated transformation of Arabidopsis thaliana. Plant J.: Cell Mol. Biol..

[38] Ross KJ, Fransz P, Jones GH (1996). A light microscopic atlas of meiosis in Arabidopsis thaliana. Chrom. Res..

[39] Mozo T, Fischer S, Meier-Ewert S, Lehrach H, Altmann T (1998). Use of the IGF BAC library for physical mapping of the Arabidopsis thaliana genome. Plant J.: Cell Mol. Biol..

[40] Choi S, Creelman RA, Mullet JE, Wing RA (1995). Construction and characterization of a bacterial artificial chromosome library ofArabidopsis thaliana. Plant Mol. Biol. Rep..

[41] Lysak M, Fransz P, Schubert I (2006). Cytogenetic analyses of Arabidopsis. Methods Mol. Biol. (Clifton, N. J.).

