# Supplementary material for: Changing local recombination patterns in Arabidopsis by CRISPR/Cas mediated chromosome engineering
Source: Nat Commun. 2020 Sep 4;11:4418. doi: 10.1038/s41467-020-18277-z (PMC7474074; doi:10.1038/s41467-020-18277-z)
Supplement: Supplementary file 1 — Supplementry Information [file 41467_2020_18277_MOESM1_ESM.pdf]

Changing local recombination patterns in Arabidopsis by CRISPR/Cas mediated chromosome engineering

Schmidt *et al.*

Supplementary Table 1. List of used oligonucleotides.

Name of oligonucleotide	Sequence 5‘-3‘
Spacer rknob d FW	attgCACTAAAGAGGGGTGGGGAG
Spacer rknob d REV	aaacCTCCCCACCCCTCTTTAGTG
Spacer rknob p FW	attgATAATGAGACGATGGGTTTG
Spacer rknob p REV	aaacCAAACCCATCGTCTCATTAT
rknob p junction EC FW	CCGAATCCCCAATAAATGAGTTCC
rknob p junction EC REV	CCATTCTTGACGTTGTCCTCACC
rknob d junction EC FW	ACCAAATGTTGATGACGAGGCTG
rknob d junction EC REV	GCTGCCCTATCATTGCGGTTAT
rknob F1 FW	TTCACGGCGTTTCCTGTCC
rknob F1 REV	CTACATACGATGAGCGACGGC
rknob F2 FW	GCTGGGTAAACCACATTAGTGC
rknob F2 REV	CGTTAAAGGAGTTGCTAATGTCGG
rknob F3 FW	CGTACCTCCTTGGACACGAC
rknob F3 REV	CGAAGCTGAAGCAAATGTCAAAC
rknob F4 FW	GCAGAGAAGTCGAAAGAAGTTTGG
rknob F4 REV	GAAGCGGCTGGGTTTGAGAT
rknob F5 FW	GAAACTGTGAGTGAGCTTGTGGAG
rknob F5 REV	GGTAACGGACAAAGGCAGGTT
rknob F6 FW	CTACTCTGCTTGTCTCTCCTCG
rknob F6 REV	CCAATCTAACCCGAATCAGCCA
rknob F7 FW	AGGTGATTCTGATGAAGTGGTTTG
rknob F7 REV	GCTGATGCCTGTTGGGAGATTA
rknob F8 FW	CCAAACCATACTCTCCAAAGTCC
rknob F8 REV	CTGAGATGTGATAGCGGAAGAGAA
rknob F9 FW	TACACTGCACAAGTCAACCTTCC
rknob F9 REV	GTTGTTTATTCGGTGTGTGATGTCG
rknob F10 FW	ACATCAACAGATTACAACGCAAGTC
rknob F10 REV	CCTGACCCGCCAGTAGATAACT
SNP genotyping knob M1 FW	GCATCGGATTCGAGCATCTG
SNP genotyping knob M1 REV	GAGAATTGGACAACCGCAGATTG
SNP genotyping knob M2 FW	TGAAGGCCTTGCAAGTTAGA
SNP genotyping knob M2 REV	CAGAAGCATATATACATACCACATAACC
SNP genotyping knob M3 FW	GGATGTCTTGTAGGTTGTGTGAG
SNP genotyping knob M3 REV	TCTGGTCTGAGATTCTAGCTTTC
SNP genotyping knob M4 FW	AGGTTTGCTAGCCGGTATTG
SNP genotyping knob M4 REV	GACGCGGATCGTCTTTCTAAG
SNP genotyping knob M5 FW	ATCGTCAGGGAGGAGAGTG
SNP genotyping knob M5 REV	CCATCAAGAAGGTCAAAGTATCG
SNP genotyping knob M6 FW	GGCACATATCTCCTCCCA
SNP genotyping knob M6 REV	GCGTCAGAAGAAGTCACAAG
SNP genotyping knob M7 FW	CTTTTCCGCCTAAGGCTCG
SNP genotyping knob M7 REV	TAAAGA[+G]GAGTCGAATGAGATG

Listed are names and sequences of oligonucleotides. In the cases where LNAs were used, the corresponding nucleotide is marked with a plus sign and a square bracket.

Supplementary Table 2. Used probes.

Probe name	Sequence 5'-3'
Probe knob M1 Col-0	[HEX]CAACATTAGACCA[+T]TTCTCATATAGCTGC[BHQ1]
Probe knob M1 L*er*-1	[FAM]CAACATTAGACCA[+C]TTCTCATATAGCTGC[BHQ1]
Probe knob M2 Col-0	[HEX]GATAAAGCAGTGTTCTCTAAGATTTAAGATGAATA[BHQ1]
Probe knob M2 L*er*-1	[FAM]GATAAAGCAGTGTTCTCTGAGATTTAAGATGAATA[BHQ1]
Probe knob M3 Col-0	[HEX]GATTCAAAGATGGTGTTTGTC[+T]TCTATC[BHQ1]
Probe knob M3 L*er*-1	[FAM]GATTCAGAGATGGTGTTTGTC[+C]TCTATC[BHQ1]
Probe knob M4 Col-0	[HEX]CAGCCATACTGATGACCATGTCCAAATTTG[BHQ1]
Probe knob M4 L*er*-1	[FAM]CAGCCATACTGATGAGCATGTCCAAATTTG[BHQ1]
Probe knob M5 Col-0	[HEX]GGAGTAGATGTAGC[+G]AGTGCTGTTGATG[BHQ1]
Probe knob M5 L*er*-1	[FAM]GGAGTAGATGTAGC[+A]AGTGCTGTTGATG[BHQ1]
Probe knob M6 Col-0	[HEX]CTTATCAACTTTCGGAAGTACTGGACCTGA[BHQ1]
Probe knob M6 L*er*-1	[FAM]CTTATCAACTTCCGGAAGTACTAGACCTGA[BHQ1]
Probe knob M7 Col-0	[HEX]CTTGTTGGCTGCGTCCAGGTCCTTC[BHQ1]
Probe knob M7 L*er*-1	[FAM]CTTGTTGCCTGCTTCCAGGTCCTTC[BHQ1]

Listed are all probes used in this study. If LNAs were integrated into the probe, the corresponding base was placed in square brackets and marked with a plus sign. The fluorophore at the 5' end is marked in red and the quencher at the 3' end in blue. Nucleotides that differ between Col-0 and L*er*-1 are marked in green.

Supplementary Table 3. Mendelian segregation of the newly formed junctions of CS1233 #20.

Line	Tested plants	Hetero-zygous	Homo-zygous	Wild type	E_{hetero}	E_{Homo}	E_{WT}	B_i^2/E_i hetero	B_i^2/E_i homo	B_i^2/E_i wildtyp	X^2	Mendelian segregation
#20	40	26	8	6	20	10	10	33.8	6.4	3.6	3.8	yes
	200	102	51	47	100	50	50	104.0	52.0	44.2	0.2	yes

The T_2 generation of the line #20 was examined for the presence of the inversion-specific junctions and for the presence of wild-type junctions using PCR-based genotyping. Initially, 40 and then 200 plants were tested and a Mendelian segregation could be observed for the newly induced inversion. Using the χ^2 test with the critical value χ^2 (1; 0.95), it was determined whether the number of homozygous, heterozygous and wild typical plants corresponds to Mendel's law of segregation.

Supplementary Table 4. Mendelian segregation of the induced inversion in the T_3.

Line	Tested plants	Hetero-zygous	Homo-zygous	Wild type	E_{hetero}	E_{Homo}	E_{WT}	B_i^2/E_i hetero	B_i^2/E_i homo	B_i^2/E_i wildtyp	X^2	Mendelian segregation
#7 (38)	20	13	3	4	10	5	5	16.9	1.8	3.2	1.9	yes
#15 (35)	20	9	6	5	10	5	5	8.1	7.2	5.0	0.3	yes
#26 (12)	20	11	7	2	10	5	5	12.1	9.8	0.8	2.7	yes
#26 (19)	20	13	5	2	10	5	5	16.9	5.0	0.8	2.7	yes
#27 (18)	20	13	4	3	10	5	5	16.9	3.2	1.8	1.9	yes

Progeny of five of the positively screened T_2 lines was tested for the presence of inversion-specific and wild-type junctions using PCR-based genotyping. Using the χ^2 test with the critical value χ^2 (1; 0.95), Mendelian segregation of the inversion could be demonstrated for each tested line.

Supplementary Table 5. Overview of location and size of the amplified rknob fragments.

Fragment	Location on chromosome 4	Size (bp)
F1	1671805-1673479	1674
F2	1746375-1748271	1896
F3	1908835-1910237	1402
F4	2011619-2013175	1556
F5	2124323-2126245	1922
F6	2232842-2234194	1352
F7	2339394-2340779	1385
F8	2449626-2451299	1673
F9	2567018-2568900	1882
F10	2672059-2674188	2129

Listed are the different fragments with the corresponding size and their associated location.

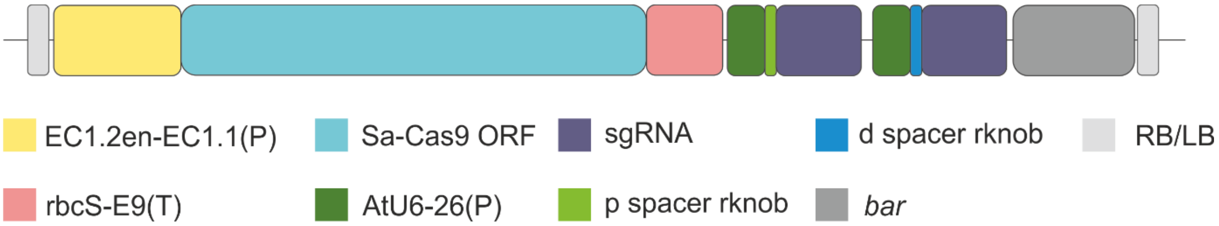

Supplementary Figure 1. Schematic representation of the final expression construct. Both spacer sequences were integrated into the pDe-Sa-Cas9 vector and finally the EC-specific promoter and the rbcS-E9 terminator were also incorporated into this vector.

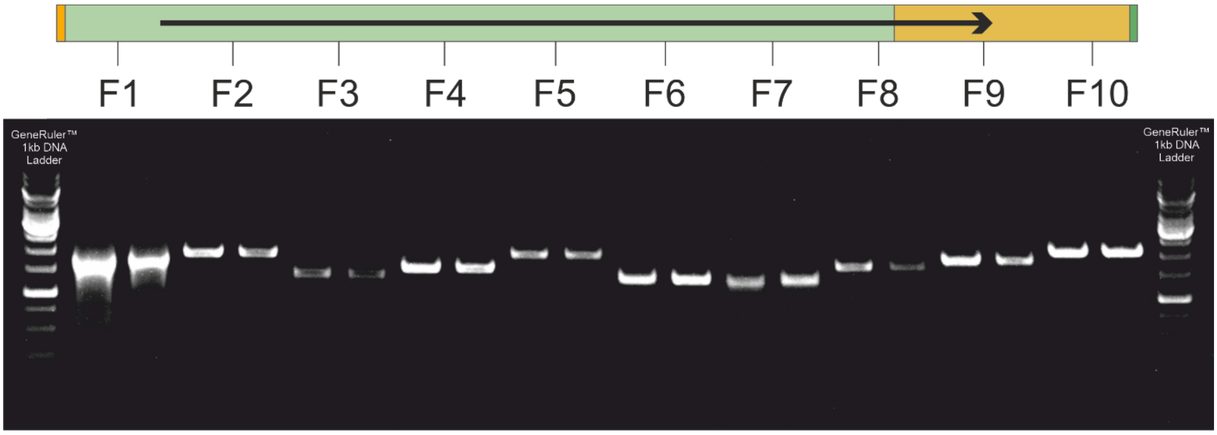

Supplementary Figure 2. Overview of the amplification of ten different fragments equally distributed over the newly induced inversion. Successful amplification could be achieved for all ten fragments and the result of the amplification from the homozygous line #20 (left) and the WT (right) was plotted for each fragment (F1-F10). Each PCR was established by using three independent leaf samples from wild type plants and afterwards amplification of each fragment was performed once with the respective lines. The size marker used was the GeneRuler™ 1kb DNA Ladder (Thermo Fisher Scientific).
