# Supplementary material for: Changing local recombination patterns in Arabidopsis by CRISPR/Cas mediated chromosome engineering
Source: Nat Commun. 2020 Sep 4;11:4418. doi: 10.1038/s41467-020-18277-z (PMC7474074; doi:10.1038/s41467-020-18277-z)
Supplement: Supplementary file 2 — Reporting Summary [file 41467_2020_18277_MOESM2_ESM.pdf]

nature research

Corresponding author(s): Holger Puchta

Last updated by author(s): Aug 7, 2020

Reporting Summary

Nature Research wishes to improve the reproducibility of the work that we publish. This form provides structure for consistency and transparency in reporting. For further information on Nature Research policies, see our Editorial Policies and the Editorial Policy Checklist.

Statistics

For all statistical analyses, confirm that the following items are present in the figure legend, table legend, main text, or Methods section.

n/a	Confirmed	
☐	☒	The exact sample size (*n*) for each experimental group/condition, given as a discrete number and unit of measurement
☐	☒	A statement on whether measurements were taken from distinct samples or whether the same sample was measured repeatedly
☒	☐	The statistical test(s) used AND whether they are one- or two-sided<br>*Only common tests should be described solely by name; describe more complex techniques in the Methods section.*
☒	☐	A description of all covariates tested
☒	☐	A description of any assumptions or corrections, such as tests of normality and adjustment for multiple comparisons
☒	☐	A full description of the statistical parameters including central tendency (e.g. means) or other basic estimates (e.g. regression coefficient) AND variation (e.g. standard deviation) or associated estimates of uncertainty (e.g. confidence intervals)
☒	☐	For null hypothesis testing, the test statistic (e.g. *F*, *t*, *r*) with confidence intervals, effect sizes, degrees of freedom and *P* value noted<br>*Give P values as exact values whenever suitable.*
☒	☐	For Bayesian analysis, information on the choice of priors and Markov chain Monte Carlo settings
☒	☐	For hierarchical and complex designs, identification of the appropriate level for tests and full reporting of outcomes
☒	☐	Estimates of effect sizes (e.g. Cohen's *d*, Pearson's *r*), indicating how they were calculated

Our web collection on statistics for biologists contains articles on many of the points above.

Software and code

Policy information about availability of computer code

Data collection	All sequence information was obtained from TAIR (https://www.arabidopsis.org/) and Arabidopsis 1,001 Genome Browser (http://signal.salk.edu/atg1001/3.0/gebrowser.php). Sanger sequencing was performed by GATC Eurofins. Microscopy slides were examined with a Zeiss AxioScope A1 fluorescence microscope using small band pass filters for DAPI, FITC and Cy3. Selected images were captured with a Nikon color DS-Ri2 camera using Nikon NIS-elements 4.60 software.
Data analysis	For evaluation of qPCR we used the LightCycler 1.5 Software (Roche). ApE (v2.0.55) was used for alignment and analysis of Sanger sequencing data. Brightness and contrast of gel electrophoresis pictures were adjusted in Corel Draw 2019 (Version 21.0.0.593). Graphs were made with Excel 2016 and CorelDraw 2019 (Version 21.0.0.593). Microscope images were further processed with Adobe Photoshop software.

For manuscripts utilizing custom algorithms or software that are central to the research but not yet described in published literature, software must be made available to editors and reviewers. We strongly encourage code deposition in a community repository (e.g. GitHub). See the Nature Research guidelines for submitting code & software for further information.

Data

Policy information about availability of data

All manuscripts must include a data availability statement. This statement should provide the following information, where applicable:

- Accession codes, unique identifiers, or web links for publicly available datasets
- A list of figures that have associated raw data
- A description of any restrictions on data availability

The authors declare that the data supporting the findings are available within the paper and its Supplementary Information, or are available from the corresponding author upon reasonable request.

Field-specific reporting

Please select the one below that is the best fit for your research. If you are not sure, read the appropriate sections before making your selection.

- [x] Life sciences
- [ ] Behavioural & social sciences
- [ ] Ecological, evolutionary & environmental sciences

For a reference copy of the document with all sections, see nature.com/documents/nr-reporting-summary-flat.pdf

Life sciences study design

All studies must disclose on these points even when the disclosure is negative.

Sample size	No sample-size calculation was performed. To determine plants harbouring an inversions rather large sample sizes were chosen, because we expected very low inversion frequencies and to randomize bias from integration of the Cas9 nuclease. As previously published in Schmidt, C., Pacher, M. & Puchta, H. Efficient induction of heritable inversions in plant genomes using the CRISPR/Cas system. The Plant journal : for cell and molecular biology 98, 577–589; 10.1111/tpj.14322 (2019). the inversion frequency ranges between 0.5 and 2 % with a distance of up to 18 kb. We decided to screen approximately 1600 plants to be able to detect inversions that occur with a frequencies of about 0.1 %.<br>For the F1 pollen analysis we tested all pollen for which the WGA was successful. In the F2 generation we tested up to 200 individual plants and selected this number based on previously detected crossover frequencies in Arabidopsis (CO were detected in 6 regions with an average size of 240 kb).
Data exclusions	In F1 pollen analysis we excluded the CO frequency of I5, because no CO event could be detected in this area due to low sample size in F1. We were able to show occurence of COs in I5 in the F2 generation with appropriate sample size.
Replication	For screening of heritable inversions, 1600 independent plants were analysed via PCR-based genotyping and these experiments were always carried out analysing 3 technical replicates for each junction primer combination. After a positive result was obtained, new leaf material was taken from the respective plant again analysed with 3 technical replicates. For F1-hybrid recombination analysis (after backcrossing) we analysed all pollen that passed the quality control of WGA (54-90 plants) and these results were verified in the corresponding F2-Hybrid analysis (200 individual randomly choosen F2-Hybrids) marker analysis with this material was done as duplicates.
Randomization	All independent transgenic lines and individuals plants were randomly picked for analysis.
Blinding	Not required for most analyses, as samples were processed identically through standard procedures that should not bias outcomes. As plants were always numbered randomly during line establishment and for analysis blinding was not relevant to our study.

Reporting for specific materials, systems and methods

We require information from authors about some types of materials, experimental systems and methods used in many studies. Here, indicate whether each material, system or method listed is relevant to your study. If you are not sure if a list item applies to your research, read the appropriate section before selecting a response.

Materials & experimental systems

n/a	Involved in the study	
x		Antibodies
x		Eukaryotic cell lines
x		Palaeontology and archaeology
x		Animals and other organisms
x		Human research participants
x		Clinical data
x		Dual use research of concern

Methods

n/a	Involved in the study	
x		ChIP-seq
	x	Flow cytometry
x		MRI-based neuroimaging

Flow Cytometry

Plots

Confirm that:

- [ ] The axis labels state the marker and fluorochrome used (e.g. CD4-FITC).
- [ ] The axis scales are clearly visible. Include numbers along axes only for bottom left plot of group (a 'group' is an analysis of identical markers).
- [ ] All plots are contour plots with outliers or pseudocolor plots.
- [ ] A numerical value for number of cells or percentage (with statistics) is provided.

Methodology

Sample preparation	Pollen of Arabidopsis were grinded in nuclei isolation buffer (Galbraith et al. 1983) with 4mm steel balls using a Retsch mill (20 s<br>at 30 hz/s), filtered through a 20µm mesh and stained with PI (50 µg/ml)
Instrument	BD Influx Cell Sorter (BD Biosciences)
Software	BD FACS Sortware 1.2.0.142 (BD Biosciences)
Cell population abundance	Pollen nuclei were differentiated from the debries using a dot plot DNA fluorescence (PI) versus Forward Scatter (FSC), Sorting was performed using the '1.0 drop single' mode to achieve the highest purity
Gating strategy	The nuclei population was first gated in a PI-fluorescence-log/FSC-log dot plot and subsequently the sorting gate defined in a PI-fluorescence-lin histogram

- [ ] Tick this box to confirm that a figure exemplifying the gating strategy is provided in the Supplementary Information.
