# Supplementary figures and images for: Changing local recombination patterns in Arabidopsis by CRISPR/Cas mediated chromosome engineering
Source: Nat Commun. 2020 Sep 4;11:4418. doi: 10.1038/s41467-020-18277-z (PMC7474074; doi:10.1038/s41467-020-18277-z)

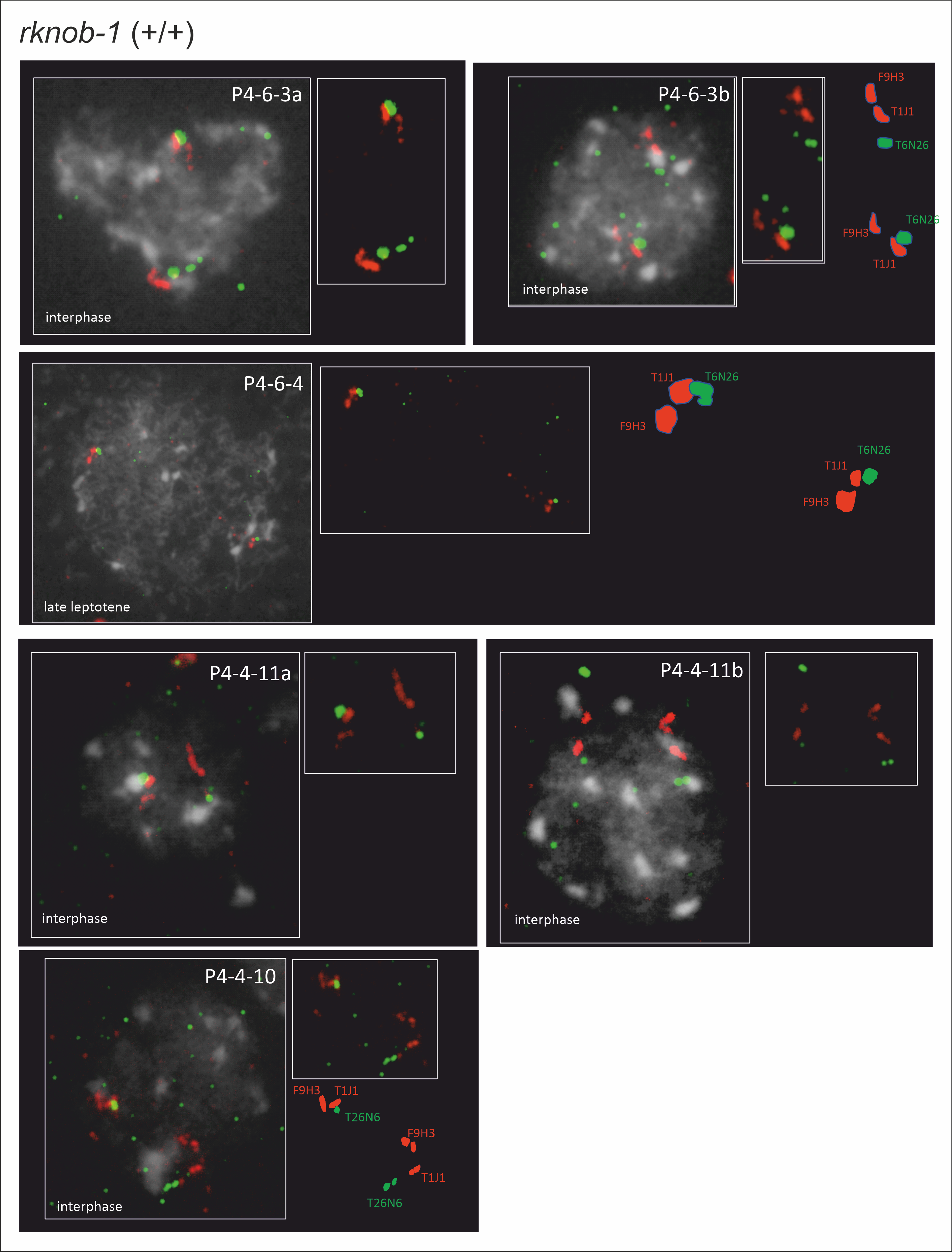

Supplement: Supplementary file 3 — Source Data [file 41467_2020_18277_MOESM3_ESM.zip › SourceData Fish rknob-1.png]

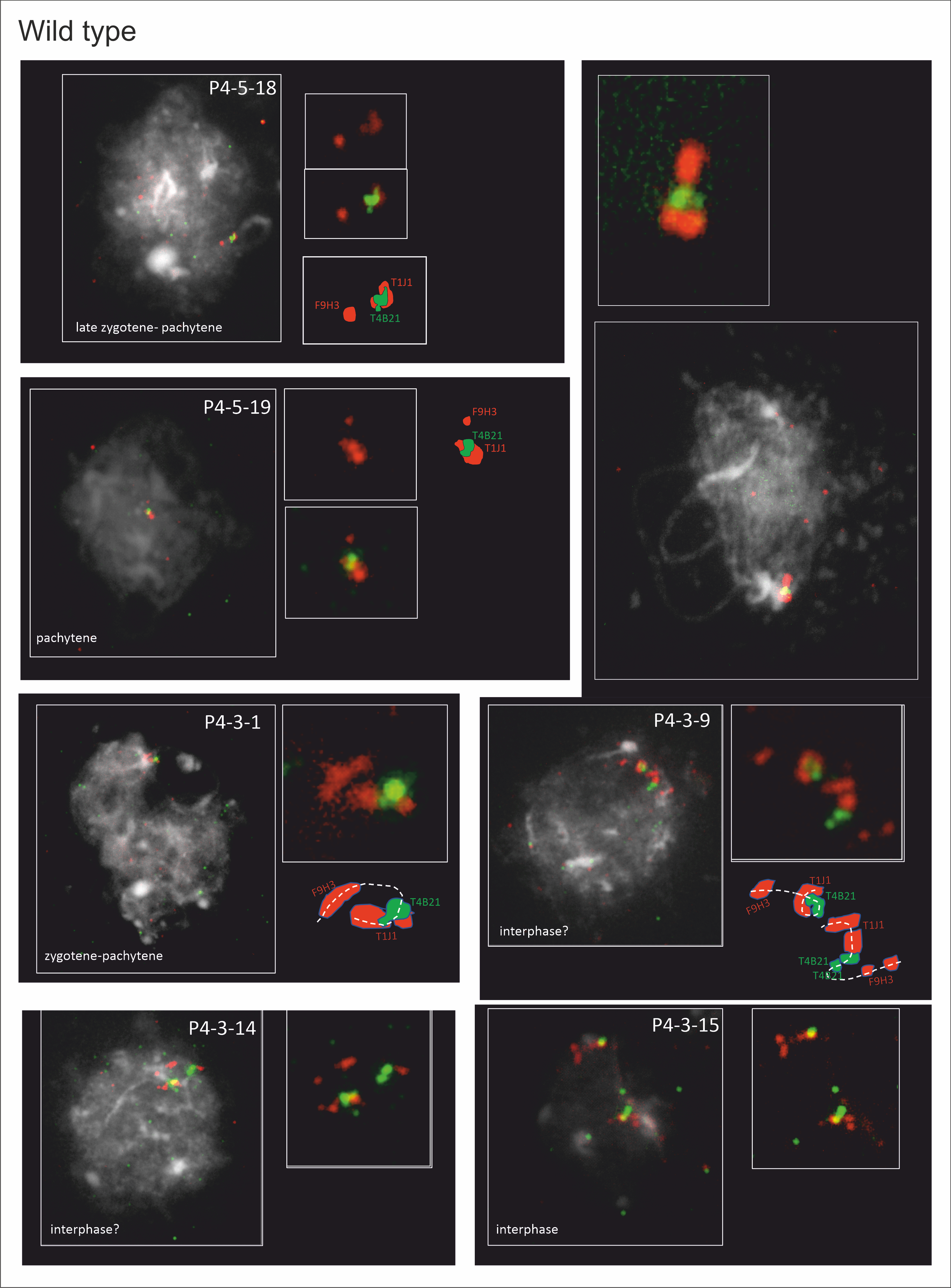

Supplement: Supplementary file 3 — Source Data [file 41467_2020_18277_MOESM3_ESM.zip › SourceData Fish wild type.png]
